# The utility of first trimester plasma glycated CD59 (pGCD59) in predicting gestational diabetes mellitus: A prospective study of non-diabetic pregnant women in Ireland

**DOI:** 10.1016/j.diabres.2022.110023

**Published:** 2022-08

**Authors:** Delia Bogdanet, Michelle Toth Castillo, Helen Doheny, Louise Dervan, Miguel Angel Luque-Fernandez, Jose Halperin, Paula M. O'Shea, Fidelma P. Dunne

**Affiliations:** a College of Medicine, Nursing and Health Sciences, School of Medicine, National University of Ireland, Galway, Ireland; b Divisions of Haematology, Brigham & Women’s Hospital, Harvard Medical School, United States; c Department of Clinical Biochemistry, Saolta University Health Care Group (SUHCG), Galway University Hospitals, Galway, Ireland; d Department of Epidemiology, Harvard T.H. Chan School of Public Health, Boston, MA, United States; e Department of Epidemiology and Population Health, London School of Hygiene and Tropical Medicine, London, UK

**Keywords:** Gestational diabetes mellitus, Biomarker, Prediction

## Abstract

**Aims:**

To evaluate the ability of first trimester plasma glycated CD59 (pGCD59) to predict gestational diabetes mellitus (GDM) at 24–28 weeks of gestation.

**Methods:**

Prospectively, in 378 pregnant women, GDM was diagnosed using the one step 2 h 75 g oral glucose tolerance test adjudicated by the World Health Organisation (WHO) 2013 criteria. The ability of pGCD59 to predict GDM was assessed using receiver operating characteristic (ROC) curves adjusted for maternal age, body mass index (BMI), maternal ethnicity, parity, previous GDM, family history of diabetes mellitus and week of gestation at time of pGCD59 sampling.

**Results:**

pGCD59 generated an adjusted area under the curve (AUC) of (a) 0.63 (95 %CI:0.56–0.70, p < 0.001) for predicting GDM, and (b) 0.71 (95 %CI:0.62–0.79, p < 0.001 for GDM diagnosed with a fasting plasma glucose (FPG) ≥ 5.1 mmol/L. Sensitivity analysis of BMI subgroups showed that pGCD59 generated the highest AUC in the 35 kg/m^2^ ≤ BMI < 40 kg/m^2^ (AUC:0.85, 95 %CI:0.70–0.98) and BMI ≥ 40 kg/m^2^ (AUC:0.88, 95 %CI:0.63–0.99) categories.

**Conclusions:**

Early in pregnancy, pGCD59 may be a good predictor of GDM in women with a high BMI and a fair predictor of GDM diagnosed by an elevated FPG independent of BMI.

## 1 Introduction

Gestational Diabetes Mellitus (GDM) is a common pregnancy complication associated with increased morbidity and mortality for the mother and her child.

GDM is ordinarily diagnosed at the end of the second trimester of pregnancy (24–28 weeks of gestation (WG)) by an oral glucose tolerance test (OGTT) alone or preceded by a glucose challenge test (GCT). Currently there is insufficient evidence to recommend early testing [1] but it is hypothesized that early identification of women with or at risk of GDM would allow more timely intervention through increased follow-up appointments, early lifestyle modifications by improving diet and exercise levels and potentially early initiation of pharmacological treatment (metformin, insulin etc) overall decreasing foetal exposure to hyperglycaemia and leading to a reduction in adverse pregnancy outcomes. Evidence from large randomised control trials (RCTs) is needed to assess if earlier identification of women with early GDM or at risk of developing GDM, might improve maternal and neonatal pregnancy outcomes [2].The National Institute of Diabetes and Digestive and Kidney Diseases (NIDDK) has highlighted the need for level I evidence on the topic of benefits of early screening [3]. However, at present, the current glucose thresholds used for GDM diagnosis have not been validated for early screening and there is no alternative accepted method for early identification of women at risk of developing GDM.

CD59 is a complement regulatory protein that protects self-cells from complement mediated damage by specifically inhibiting membrane attack complex (MAC) formation [4]. In diabetes mellitus (DM), the complement regulatory function of CD59 is inhibited by the non-enzymatic glycation of the Lys^41^ amino acid residue forming a functionally inactive plasma glycated CD59 (pGCD59) leading to increased MAC deposition and cell damage. Although CD59 is a cell membrane bound protein, a soluble form of pGCD59 shed from cell membranes is found in plasma and can be measured with a highly sensitive and specific ELISA [5]. pGCD59 assessment during 2-step GDM screening revealed that pGCD59 predicted abnormal GCT results with a sensitivity of 90%, specificity of 88% and adjusted area under the curve (AUC) of 0.92 (95 %CI:0.88–0.93). Furthermore, pGCD59 predicted the 3 h 100 g OGTT failure with a sensitivity of 85%, specificity of 92% and adjusted AUC of 0.92 (95 %CI:0.77–0.91), independently of age, BMI, ethnicity of history of DM[6]. Recently, pGCD59 assessment in a high-risk population showed that pGCD59 levels taken prior to 20 WG predicted the diagnosis of GDM in early pregnancy (<20 WG) with an AUC of 0.86 [7]. This evidence supports the fact that pGCD59 is an emerging biomarker for GDM.

The aim of this study was to evaluate the ability of first trimester pGCD59 to predict the results of the 2 h 75 g OGTT at 24–28 WG, employing the World Health Organization (WHO) 2013 criteria, in an unselected population of pregnant women across all BMI categories.

## 2 Material and methods

The protocol for this study has been published [8]. In brief, this was a prospective study which recruited consecutive pregnant women attending their first antenatal visit at Galway University Hospital, Galway, Ireland between November 2018, and March 2020. Only pregnant women over 18 years of age with no established DM were invited to participate. At the first clinic appointment, the patient information leaflet was provided. For those willing to participate, a consent was signed, and the first sample of blood was drawn at the same time as the routine first-trimester blood tests.

Data on women's weight and height were measured using SECA scales model 799 (22089 Hamburg, Germany) at their first antenatal appointment and the BMI calculated and stratified according to WHO guidelines as underweight (<18.5 kg/m^2^), normal weight (18.5–24.9 kg/m^2^), overweight (25–29.9 kg/m^2^), and obese (≥30 kg/m^2^). An ultrasound scan was performed to confirm gestational age, diagnose any significant foetal defects, and assess the thickness of the foetal nuchal translucency.

All women were offered screening for GDM using a 2 h 75 g OGTT performed in the second trimester (24–28 WG). Participants were advised that the test would be performed in the morning after an overnight fast of 8–12 h and after at least 3 days of unrestricted diet and unlimited physical activity. On the day of the test, while still fasting, venous whole blood was collected into fluoride oxalate specimen tubes for glucose measurement. Each woman was then given Rapilose® OGTT Solution (Penlan Healthcare Ltd., Abbey House, Wellington Way, Weybridge, UK), a ready-to-use 300 mL pouch containing 75 g anhydrous glucose to consume within 5 min. Women were also instructed to remain seated and not to smoke, eat or drink anything for the duration of the test. Venous whole blood was collected into fluoride oxalate specimen tubes at 1 h and 2 h post ingestion of this glucose load. According to the WHO 2013 criteria, GDM was identified if one abnormal plasma glucose value was identified by the OGTT (fasting value 5.1 mmol/L (92 mg/dL), 1 h value 10 mmol/L (180 mg/dL), and 2 h value 8.5 mmol/L (153 mg/dL)) [9]. Plasma glucose was analysed using the hexokinase method on the Roche Cobas® 8000 analyser (Roche Diagnostics, Indianapolis, US). The method principle is enzymatic, utilising hexokinase which catalyses the phosphorylation of glucose to glucose-6-phosphate. The between-run analytical coefficient of variation (CVa%) for glucose at mean concentrations of 2.5 mmol/L (46 mg/dL), 7.1 mmol/L (128 mg/dL) and 16.7 mmol/L (300 mg/dL) was 1.1%, 0.9 % and 1.0%, respectively”. Assay bias was determined by proficiency testing through the United Kingdom National External Quality Assessment Scheme (UKNEQAS). The target plasma glucose concentrations ranged from 2.68 to 16.91 mmol/L and the mean bias was +1.1% (acceptance limit ± 5.0%).

At the first antenatal appointment, at the time of routine blood testing, blood (10 mL) was collected into ethylenediaminetetraacetic acid (EDTA) for pGCD59 measurement. Each gCD59 plasma sample was divided into 2 × 500 µL aliquots barcoded and stored at −80 °C. To retain participant confidentiality, all laboratory specimens were assigned a coded identity number. On completion of recruitment, an aliquot of each participant’s EDTA plasma was transported on dry ice without defreezing to the Laboratory for Translational Research, Haematology Division, Department of Medicine in Brigham and Women's Hospital, Boston, USA for pGCD59 analysis. pGCD59 was measured using the previously published enzyme-linked immunosorbent assay (ELISA) developed by Ghosh *et al.* [5].

A clinical database linked to the barcoded samples was developed and pseudo-anonymised. The constructed database contained baseline clinical information (age, weight, height, ethnicity, blood pressure, week of gestation), obstetric history (parity, gravida), lifestyle variables (smoking status, alcohol consumption) and laboratory data (OGTT results, pGCD59 concentrations, date of sampling) on each patient [8].

### 2.1 Statistical analysis

Patient characteristics were described using mean and standard deviations/median and interquartile range for continuous variables and count/percentages for categorical variables. We compared baseline characteristics of pregnant women who had a normal glucose tolerance (NGT) with characteristics of women who developed GDM using crosstabulations and χ^2^ test for categorical variables, Wilcoxon-Mann-Whitney test for continuous variables not normally distributed, and Student t tests for continuous variables normally distributed. The power calculation and sample size have been previously described [8].

Logistic regression models for maternal age, BMI, maternal ethnicity, parity, previous GDM, family history of DM and week of gestation at time of pGCD59 sampling were used to evaluate the association of between maternal characteristics at baseline with GDM at 24–28 WG. We derived adjusted odds ratios (ORs) and their respective type of Wald 95% confidence intervals (CI).

The ability of pGCD59 to predict the results of the 24–28 WG OGTT was assessed using unadjusted nonparametric receiver operating characteristic (ROC) curves and adjusted ROC curves for maternal age, BMI, maternal ethnicity, parity, previous GDM, family history of DM and week of gestation at pGCD59 sampling. Then, their respective AUC was derived with their respective 95% CI.

Missing data were assumed to be completely at random and a complete case analysis was performed. In all analyses, P < 0.05 was considered statistically significant. All statistical analyses were performed using SPSS for Windows, version 20 (IBM SPSS Statistics for Windows, version 20 SPSS, Chicago, IL).

### 2.2 Ethics

Ethical approval for this study was granted by the Clinical Research Ethics Committee, Galway, Ireland (Reference No- C.A. 2026).

## 3 Results

**Fig. 1:**
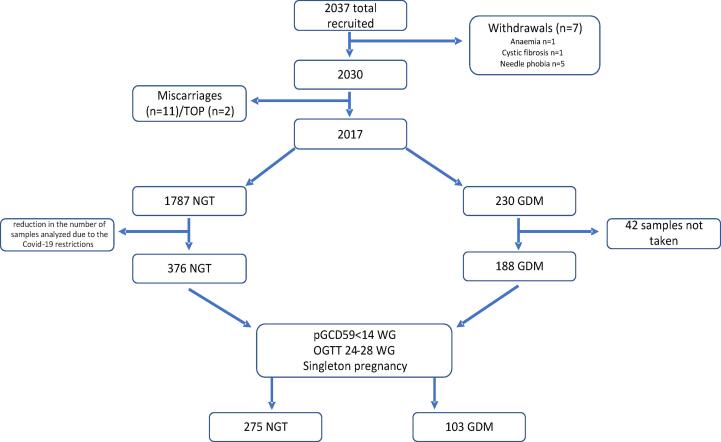
Study flowchart from women attending their first antenatal visit at Galway University Hospital, Galway, Ireland between November 2018 and March 2020.

A total of 2,037 women were recruited to this study, 7 of whom withdrew consent, 11 miscarried and 2 women underwent terminations of pregnancy (TOP). The reasons for withdrawal included: anaemia (n = 1), cystic fibrosis (n = 1) and needle phobia (n = 5) (Fig. 1). Of the remaining 2017 participants, 230 women were diagnosed with GDM which translates into a prevalence of 11.4%, which is similar to the prevalence reported for our population 10 years ago [10]. Of the 230 women with GDM, 42 did not have first trimester (T1) samples taken resulting in 188 GDM women with samples for pGCD59 analysis. We selected 376 study participants with NGT who had a sample taken at the first antenatal appointment and underwent an OGTT at 24–28 WG, resulting in a total cohort of 564 study participants. From these 564 women, we selected only those with a singleton pregnancy and the first sample for pGCD59 taken at < 14 WG (T1) with an OGTT between 24 and 28 WG (NGT n = 275, GDM n = 103) (Fig. 1).

**Fig. 2:**
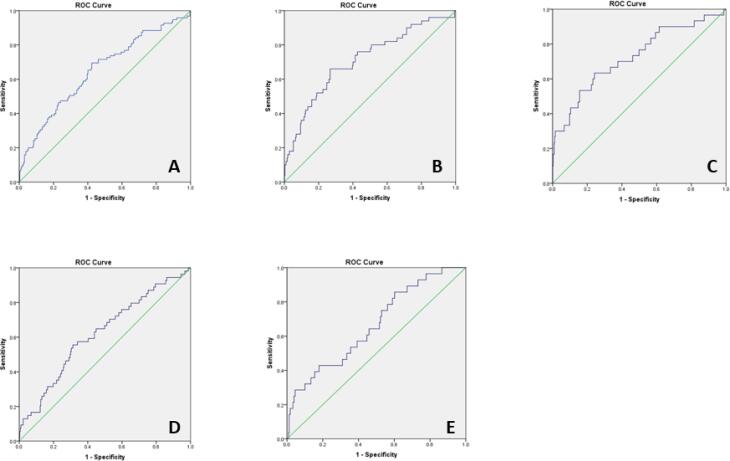
pGCD59 (<14 WG) - adjusted ROC curves for maternal age, BMI, maternal ethnicity, parity, previous GDM, family history of diabetes and week of gestation at pGCD59 sampling at Galway University Hospital, Galway, Ireland between November 2018 and March 2020, n = 378. **A:** pGCD59 prediction of GDM status AUC:0.64 95 %CI: 0.56–0.70; **B:** pGCD59 prediction of fasting glucose of 5.1 mmol/L, AUC:0.71 95 %CI: 0.62–0.79; **C:** pGCD59 prediction of fasting glucose of 5.3 mmol/L, AUC:0.73 95 %CI: 0.62–0,83; **D:** pGCD59 prediction of 1-h glucose of 10 mmol/L 95 %CI: 0.53–0.70; **E:** pGCD59 prediction of 2-h glucose of 8.5 mmol/L, AUC:0.67 95 %CI: 0.56–0.76.

Patients’ characteristics and laboratory values are described in Table 1. Women with GDM had higher systolic blood pressure (SBP) compared to women with NGT (122 vs.120 mmHg, p = 0.03) and higher mean blood pressure (BP) (88.3 vs. 86.0 mmHg, p = 0.02). There were no other differences in baseline characteristics between the two cohorts. As expected, women with GDM had higher glucose values at all time points on the OGTT (24–28 WG) (p < 0.01). T1 pGCD59 levels, however, were not different between women with GDM and women with NGT (p = 0.90) (Table 1, Fig. 2).

**Table 1:** Women’s baseline characteristics at first antenatal visit (<14 weeks gestation) and laboratory values at Galway University Hospital, Galway, Ireland between November 2018 and March 2020, n = 378.

Baseline characteristics	**NGT, n = 275** **(IQR/%)**	**GDM, n = 103** **(IQR/%)**	**P value**
Age (years)	33.6 (31.1–36.4)	34.8 (31.7–37.4)	0.07
WG at booking	12.7(12–13.1)	12.4(12–13.1)	0.60
Gravida	2(1–3)	2(1–3)	0.30
Parity	1 (0–1)	1(0–2)	0.60
Height (cm)	165(161.6–169.5)	164(160–169)	0.10
Weight (kg)	73(64–86.2)	75.7(64.2–89.6)	0.30
BMI (kg/m^2^)	26.4(23.3–31)	28.7(23.7–31.9)	0.10
Ethnicity (white)	243/275 (88.4)	88/103 (85.4)	0.60
SBP (mmHg)	120(112–126)	122(114–130)	0.03
DBP (mmHg)	69(62–75)	69(64–79)	0.10
Mean BP (mmHg)	86 (80.6–91)	88.3(79.6–94.3)	0.02
WG at delivery	40(39–40.8)	39.4(38.8–40.4)	0.07
Alcohol at booking	4/275 (1.4)	1/103 (0.9)	0.20
Alcohol before pregnancy	233/275 (84.7)	81/103 (78.6)	0.30
Non-smoker	145/275 (52.7)	55/103 (53.3)	0.90
Smoker at booking visit	14/275 (5)	8/103 (7.7)	0.20
Laboratory values			
T1pGCD59 (SPU)	3.6 (2.8–4.4)	3.7 (2.9–4.5)	0.90
OGTT 24–28 weeks:			
Fasting glucose (mmol/L)	4.4 (4.2–4.6)	5.1(4.6–5.3)	<0.01
1-h glucose (mmol/L)	7 (5.8–7.9)	10 (8.7–10.8)	<0.01
2-h glucose (mmol/L)	5.5 (4.8–6.4)	7.1 (6–8.7)	<0.01
Mean glucose (mmol/L)	5.5 (5.1–6.1)	7.2 (6–8.7)	<0.01

BMI: body mass index; BP: blood pressure; DBP: diastolic blood pressure; GDM: gestational diabetes; NGT: normal glucose tolerance; OGTT: oral glucose tolerance test; SBP: systolic blood pressure; T1: 1st trimester; WG: weeks of gestation.

Regarding risk factors for GDM, previous GDM was positively associated with the risk of GDM (OR:5.80, 95 %CI:2.1–16.1, p < 0.001). SBP and mean BP were also associated with a slight increase in GDM risk (OR:1.02, 95 %CI:1.00–1.05, p = 0.02; OR:1.03, 95 %CI:1.00–1.06, p = 0.03) (Table 2). T1 pGCD59 levels were not associated with an increased risk for GDM.

**Table 2:** GDM univariate risk factors at baseline at Galway University Hospital, Galway, Ireland between November 2018 and March 2020, n = 378.

Variables	**Odds Ratio**	**95 %CI**	**P value**
Age	1.04	0.90–1.09	0.07
Gravida	1.08	0.90–1.20	0.20
Parity	1.05	0.80–1.30	0.60
Alcohol at booking	1.06	0.40–2.60	0.80
Alcohol before pregnancy	0.80	0.70–1.10	0.40
Height	0.90	0.90–1.01	0.10
Weight	1.00	0.90–1.02	0.30
BMI	1.03	0.90–1.07	0.10
BSA	1.40	0.50–4.06	0.50
SBP	1.02	1.00–1.05	0.02
DBP	1.02	0.90–1.04	0.10
Mean BP	1.03	1.00–1.06	0.03
Non-smoker	1.03	0.60–1.60	0.90
Smoker at booking visit	1.50	0.60–3.80	0.30
Ethnicity	1.10	0.80–1.50	0.40
Family history	1.30	0.90–1.90	0.08
Previous GDM	5.80	2.10–16.1	<0.01

BMI: body mass index; BSA: body surface area; DBP: diastolic blood pressure; GDM: gestational diabetes; SBP: systolic blood pressure; T1: 1st trimester.

T1 pGCD59 generated an unadjusted AUC for predicting GDM at 24–28 WG of 0.51 (data not shown). When analysis was adjusted for maternal age, BMI, maternal ethnicity, parity, previous GDM, family history of DM and week of gestation at time of pGCD59 sampling, the AUC increased to 0.63 (95 %CI:0.56–0.70, p < 0.001) (Fig. 2, A). We further explored the ability of pGCD59 to predict GDM status on individual samples on the OGTT using the WHO 2013 diagnostic values as well as GDM diagnosis using a FPG value of 5.3 mmol/L (FPG diagnostic threshold using the ADA criteria [11]). pGCD59 predicted GDM status diagnosed with a FPG value of 5.1 mmol/L with an adjusted AUC of 0.71 (95 %CI:0.62–0.79, P < 0.001)) (Fig. 2, B), a FPG value of 5.3 mmol/L with an adjusted AUC of 0.73 (95 %CI:0.62–0.83, p < 0.001) (Fig. 2, C), a 1 h glucose value of 10 mmol/L with an adjusted AUC of 0.62 (95 %CI:0.53–0.70, p < 0.001) (Fig. 2, D), and a 2 h glucose value of 8.5 mmol/L with an adjusted AUC of 0.66 (95 %CI:0.56–0.76, p < 0.001) (Fig. 2, E).

**Fig. 3:**
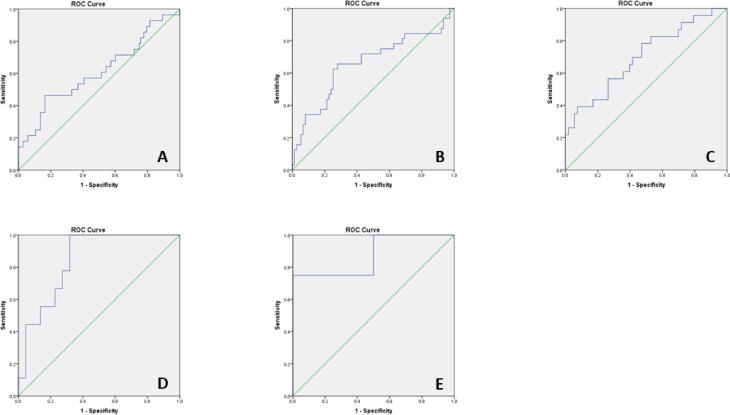
pGCD59 (<14 WG) prediction of GDM status (24–28 WG) by BMI categories - adjusted ROC curves for maternal age, BMI, maternal ethnicity, parity, previous GDM, family history of diabetes and week of gestation at pGCD59 sampling at Galway University Hospital, Galway, Ireland between November 2018 and March 2020, n = 378. **A:** BMI < 25 m/kg^2^, AUC: 0.60 95 %CI: 0.48–0.73; **B:** 25 kg/m^2^ ≤ BMI < 30 kg/m^2^, AUC: 0.65 95 %CI: 0.53–0.78; **C:** 30 kg/m^2^ ≤ BMI < 35 kg/m^2^, AUC: 0.70 95 %CI: 0.56–0.83; **D:** 35 kg/m^2^ ≤ BMI < 40 kg/m^2^, AUC: 0.85 95 %CI: 0.70–0.98; **E:** BMI ≥ 40 kg/m^2^ AUC: 0.88 95 %CI: 0.63–0.99.

A sensitivity analysis was performed to investigate the ability of T1 pGCD59 to predict GDM status according to BMI subcategories (Fig. 3). pGCD59 generated the highest AUC in women with a BMI ≥ 35 kg/m^2^ and < 40 kg/m^2^ (AUC:0.85 95 %CI:0.70–0.98) and ≥ 40 kg/m^2^ **(**AUC:0.88 95 %CI:0.63–0.99).

## 4 Discussion

In this prospective study we analysed the ability of T1 pGCD59 to predict GDM diagnosed at 24–28 WG using the 2 h 75 g OGTT and WHO 2013 criteria. We found that early pGCD59 may be a good predictor of GDM status in subjects with a high BMI and a fair predictor of GDM status diagnosed by an elevated FPG independent of BMI.

In a case-control study of 1,000 pregnant women (GDM n = 127, Carpenter and Coustan criteria [12]), Ghosh *et al.* [6] found that pGCD59 sampled at 26 WG could identify women who fail the GCT or are diagnosed with GDM with an AUC of 0.92. One explanation for the discrepancy in results between our study and the study by Ghosh *et al.* is that, in the Ghosh study, GDM was diagnosed using a 2-step process: initial screening with a 50 g GCT followed by a 3 h 100 g OGTT in cases where the GCT glucose levels > 7.8 mmol/L. In addition, two abnormal glucose values are required to make a diagnosis of GDM. This arguably selects a population of women at higher risk of GDM and may not include women with milder forms of GDM. In contrast, the women in our study were from an unselected population and screened for GDM using a 1-step 2 h 75 g OGTT, a protocol that likely selects milder cases of GDM. Furthermore, the GDM diagnostic criteria used in the study by Ghosh et al. comprised higher glucose values compared to those used in our study supporting the argument that their cohort included more severe cases of GDM compared to ours. Lastly, the timing of pGCD59 sampling was different: in our study, samples for pGCD59 was collected at < 14 WG while Gosh et al. collected samples for pGCD59 at 26 WG. This is of significance as pGCD59 is a glycated protein in which exposure to hyperglycaemia promotes the formation of GCD59 thus increasing the concentration of pGCD59 [13]. At the first antenatal visit, women diagnosed with GDM later in pregnancy may not yet have glycemia sufficiently elevated to trigger the formation of pGCD59. This would explain the lack of significant difference in pGCD59 levels between GDM and NGT women and the poor accuracy of early pregnancy pGCD59 in identifying a mid-pregnancy diagnosis of GDM. This hypothesis is supported by the higher pGCD59 levels identified in a cohort of pregnant subjects with type 1 DM (T1DM) compared to the GDM cohorts [14] as patients with T1DM have significantly higher glycaemia in early pregnancy compared to women with GDM.

A study by Ma *et al.* [7] examined the ability of pGCD59 (<20 WG) to predict GDM diagnosed < 20 WG using a 2 h 75 g OGTT employing the WHO 2013 criteria in a high-risk population (BMI ≥ 29 kg/m^2^). The authors found that pGCD59 was a good predictor of GDM diagnosed at < 20 WG with an AUC of 0.86 increasing to 0.90 when restricted to samples for pGCD59 measurement taken between 14 and 20 WG. However, the authors also found that pGCD59 < 20 WG can predict GDM at 24–28 WG (OGTT at < 20 WG normal) with an AUC of 0.68 (95 %CI: 0.64–0.73), similar to our findings. The ability of pGCD59 < 20 WG to predict GDM < 20 WG has very good accuracy in women with early GDM but poorer accuracy in women who develop GDM later in pregnancy supporting the fact that a degree of hyperglycaemia is required in order to generate pGCD59. This also might explain the poor AUC generated in our cohort as our subject participants most likely had no or minimal hyperglycaemia at < 14 WG when sampling occurred.

The impact of basal rather than reactive glycaemia on pGCD59 is reflected in our findings that pGCD59 can predict elevated FPG with fair accuracy compared to the 1 h and 2 h post glucose load plasma glucose levels. More so the prediction ability of T1 pGCD59 was higher for a FPG of 5.3 mmol/L compared to a FPG of 5.1 mmol/L at 24–28 WG. It is highly probable that women with a higher FPG at 24–28 weeks of gestation might have had a higher degree of glycaemia in the T1 of pregnancy generating higher levels of pGCD59. Longitudinal studies assessing T1 pGCD59 and T1 fasting glucose together with fasting glucose at 24–28 WG will help clarify this hypothesis.

Despite the difference in baseline average BMI between the subjects in our study and those in the Ma *et al.* study, the generated AUCs for the ability of early pGCD59 to predict 2nd trimester GDM were similar. However, on our sub analysis of BMI categories, in the very high BMI cohorts, T1 pGCD59 predicted GDM with good accuracy. This suggests the presence of a metabolic threshold above which insulin resistance generated by the adipose tissue generates a sufficient degree of glycaemia to cause the glycation of CD59 in early pregnancy. Studies evaluating the association between T1 BMI, T1 pGCD59, T1 FPG and GDM status at 24–28 WG will further elucidate the impact that adipose tissue has on early pregnancy glycaemic levels and T1 pCD59 glycation, on the changes in FPG levels between the first and second trimester of pregnancy and, implicitly, on pGCD59's ability to detect hyperglycaemia in a high BMI population.

Our study has several limitations. The Covid-19 pandemic has had a significant impact on the scientific world, including this study. The recurrent lockdowns, closure and reopening of laboratories, delays in procurement of laboratory consumables and limited availability of staff compelled us to review and amend the study design. This resulted in study deviations from the original published protocol [8] and a reduction in the number of samples analysed. Another limitation is our lack of ethnic diversity which reflects our existing population, but which reduces the generalizability of our findings. Furthermore, we did not have any data on gestational weight gain. Also, the results of our sensitivity analysis must be interpreted with caution due to the small number of cases in each BMI subcategory. An OGTT performed at the same time as the pGCD59 (<14WG) would have provided valuable information into the pathophysiology of pGCD59 in GDM, but this was beyond the scope of this study.

This study has shown that, while the overall ability of early pregnancy pGCD59 to predict GDM at 24–28 WG is poor, pGCD59 may predict an abnormal fasting glucose at 24–28 WG with fair accuracy. Furthermore, in a cohort of women with a very high BMI, early pGCD59 could identify GDM cases with very good accuracy. Future studies in larger, more ethnically diverse cohorts are required to explore the ability of early pGCD59 to predict GDM diagnosed at standard of care screening for GDM.

## CRediT authorship contribution statement

**D. Bogdanet:** Conceptualization, data curation, formal analysis, funding acquisition, Investigation, Methodology, Project administration, resources, software, visualization, writing original draft, review and editing. **M Tooth Castillo:** Data curation, formal analysis, investigation, resources, writing review and editing. **H Doheny:** Conceptualization, data curation, formal analysis, methodology, resources, software, writing review and editing. **MA Luque-Fernandez:** Data curation, formal analysis, investigation, methodology, project administration, resources, software, supervision, validation. **J Halperin:** Conceptualization, data curation, formal analysis, funding acquisition, investigation, methodology, project administration, resources, supervision, validation, visualization, writing review and editing. **P O’Shea:** Conceptualization, data curation, formal analysis, funding acquisition, investigation, methodology, project administration, resources, software, supervision, validation, visualization, writing review and editing. **F Dunne:** Conceptualization, funding acquisition, investigation, methodology, project administration, resources, supervision, validation, visualization, writing review and editing.

## Declaration of Competing Interest

The authors declare the following financial interests/personal relationships which may be considered as potential competing interests: JAH has a financial interest in Mellitus LLC. Mellitus has licensed intellectual property for the technology used in this research and in developing diagnostic tools for diabetes. The interests of J.A.H. are reviewed and are managed by Brigham and Women's Hospital and Partners HealthCare in accordance with their conflict-of-interest policies. Reagents and laboratory analysis for pGCD59 were provided by Prof Jose Halperin.
